# Supplementary figures and images for: Phytophthora infestans RXLR-WY Effector AVR3a Associates with Dynamin-Related Protein 2 Required for Endocytosis of the Plant Pattern Recognition Receptor FLS2
Source: PLoS One. 2015 Sep 8;10(9):e0137071. doi: 10.1371/journal.pone.0137071 (PMC4562647; doi:10.1371/journal.pone.0137071)

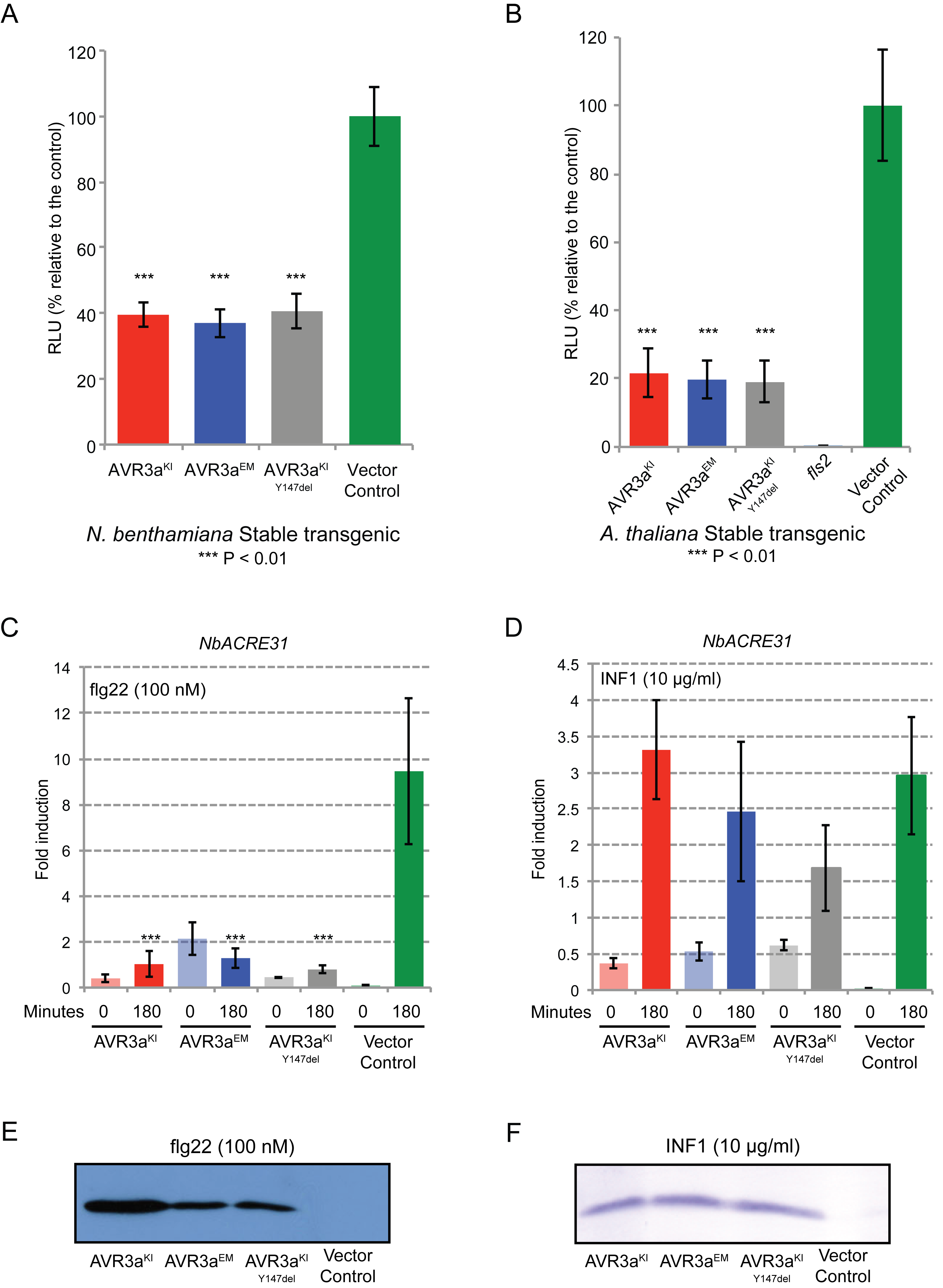

Supplement: S1 Fig — (A, B) Total ROS production measured in relative light units (RLU) is expressed as percentage of the control treated with 100 nM flg22 over 45 minutes in transgenic plants of N. benthamiana (A) or A. thaliana (B). Plants were stably transformed with the following constructs: FLAG-AVR3aKI (red), FLAG-AVR3aEM (blue), FLAG-AVR3aKI-Y147del (grey) or vector control (ΔGFP) (green). Values are average ± SE (n = 24). Statistical significance was evaluated in comparison to the control by one-way ANOVA followed by TukeyHSD test. (C, D) Expression of the marker gene NbACRE31 was assessed by qRT-PCR at time 0 and 180 minutes after elicitation with 100 nM flg22 (C) or 10 μg/ml INF1[Pi] (D) and normalized by NbEF1α gene expression. Results are average ± SE (n = 3 technical replicates). AVR3a variants were transiently expressed in N. benthamiana using the following constructs: FLAG-AVR3aKI (red), FLAG-AVR3aEM (blue), FLAG-AVR3aKI-Y147del (grey) or vector control (ΔGFP) (green). (E, F) Western blots probed with anti-FLAG antibody after flg22 (E) or INF1 (F) treatment detected total protein expression of AVR3a variants. (TIF) [file pone.0137071.s001.tif]

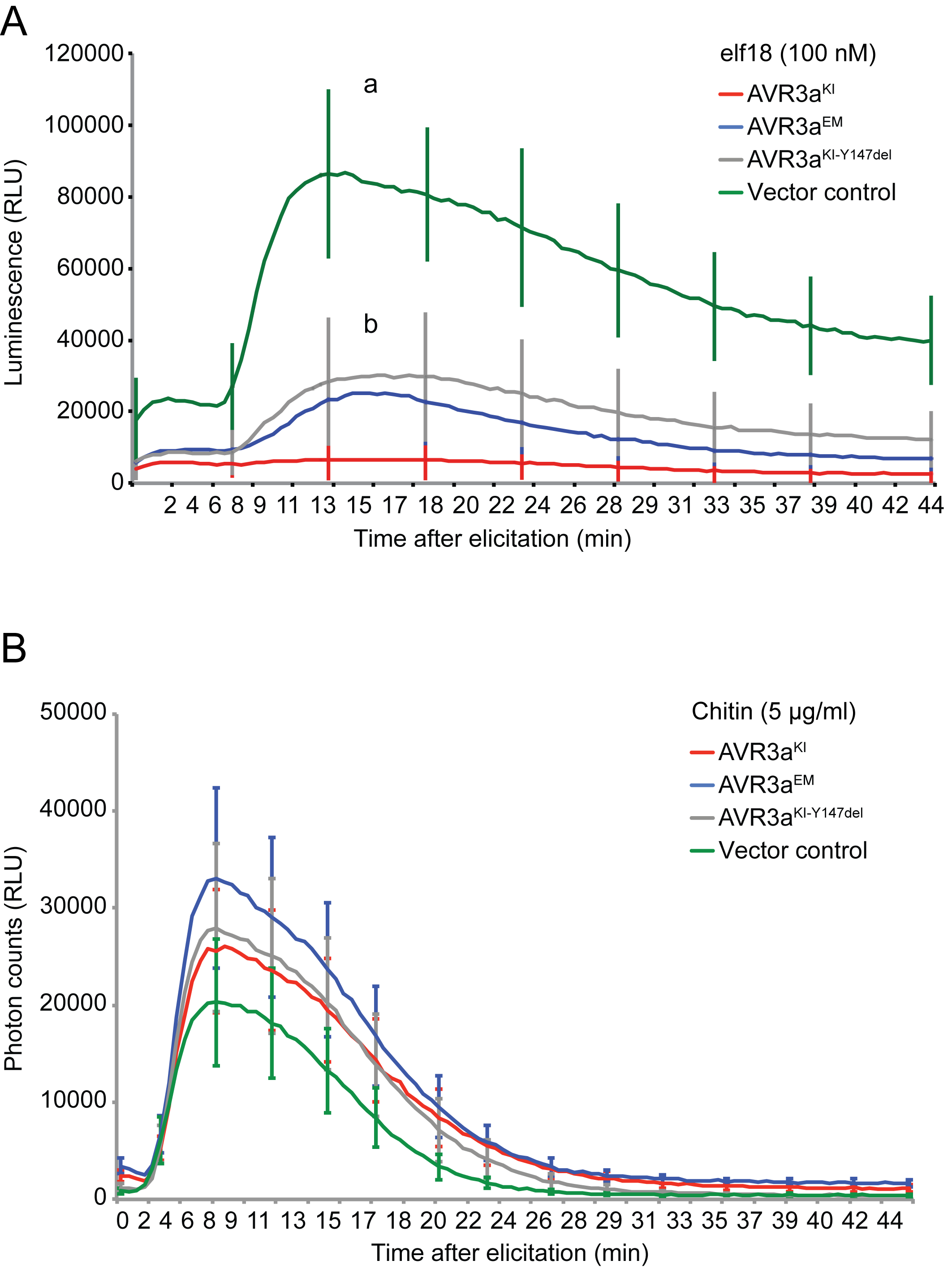

Supplement: S2 Fig — (A, B) N. benthamiana agro-infiltrated with FLAG-AVR3aKI (red), FLAG-AVR3aEM (blue), FLAG-AVR3aKI-Y147del (grey) or vector control (ΔGFP) (green). Leaf discs were incubated in an elf18 (A) or chitin (B) containing solution and ROS production was measured in relative light units (RLU) over time. Letters above the graph indicate statistical significant differences at P < 0.05 assessed by one-way ANOVA followed by TukeyHSD test. No statistical significance was found for group b (P = 0.068). Similar results were observed in two independent experiments. (TIF) [file pone.0137071.s002.tif]

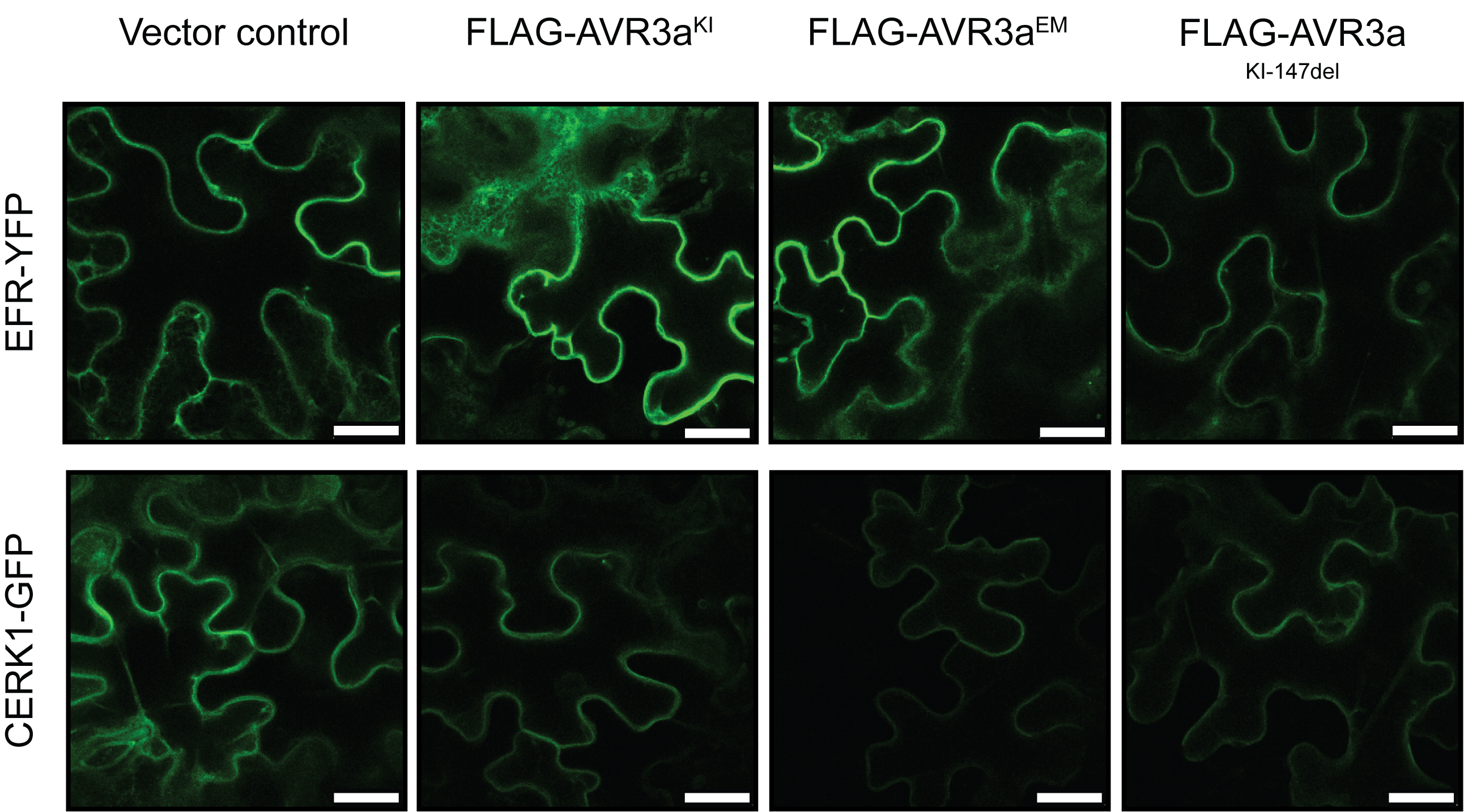

Supplement: S3 Fig — Transient co-expression at 2.5 days post infiltration in N. benthamiana of EFR-YFP-HA or CERK1-GFP with FLAG-AVR3aKI or FLAG-AVR3aEM or FLAG-AVR3aKI-Y147del or vector control (ΔGFP) as indicated. Confocal microscopy shows that the plasma membrane localization of EFR-YFP or CERK1-GFP was not altered by the presence of variants of AVR3a. Bar = 25 μm. (TIF) [file pone.0137071.s003.tif]

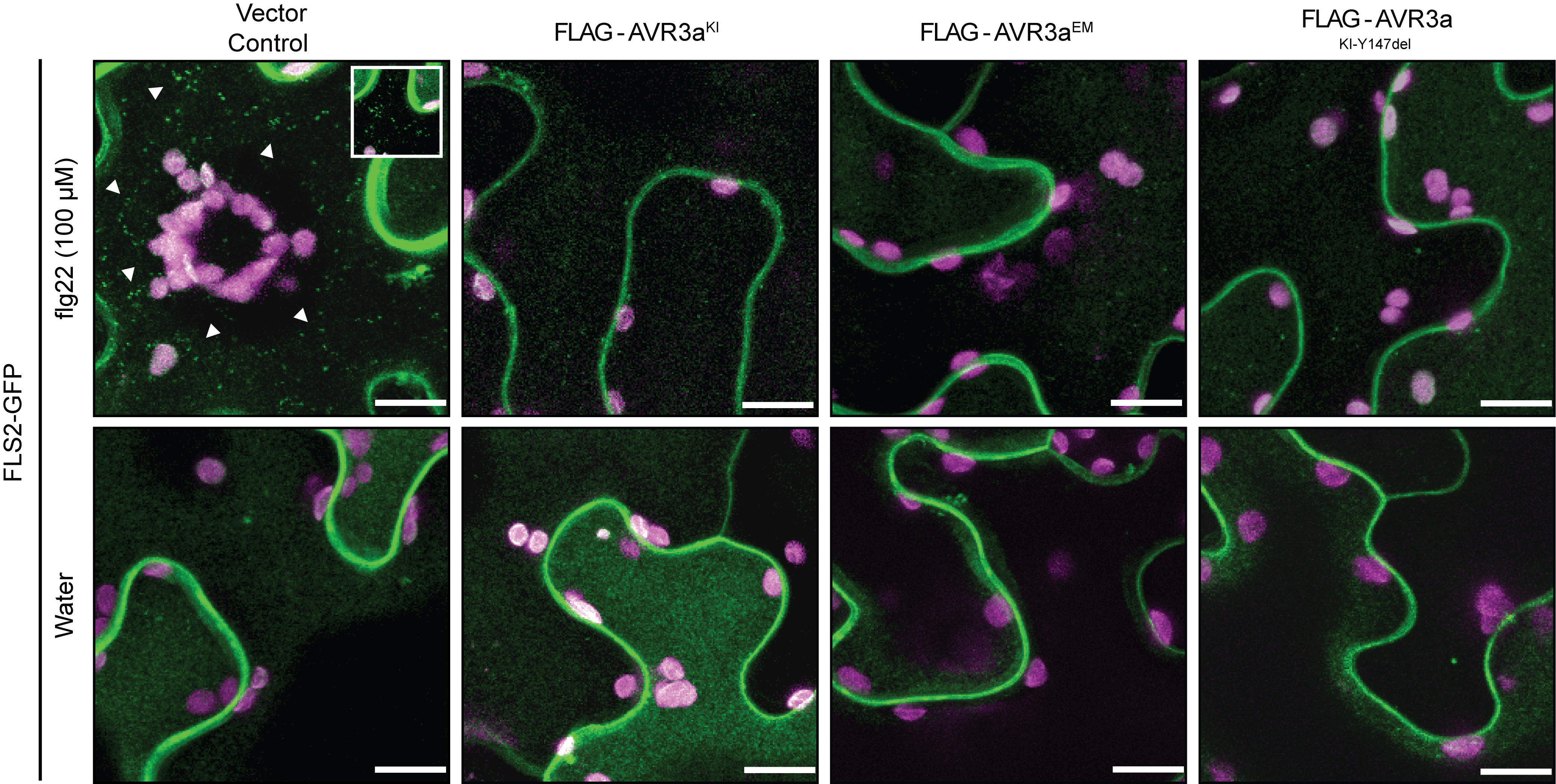

Supplement: S4 Fig — Enlarged confocal images showed in Fig 3. White arrowheads and inset image indicate FLS2 endosomes. Bar = 25 μm. (TIF) [file pone.0137071.s004.tif]

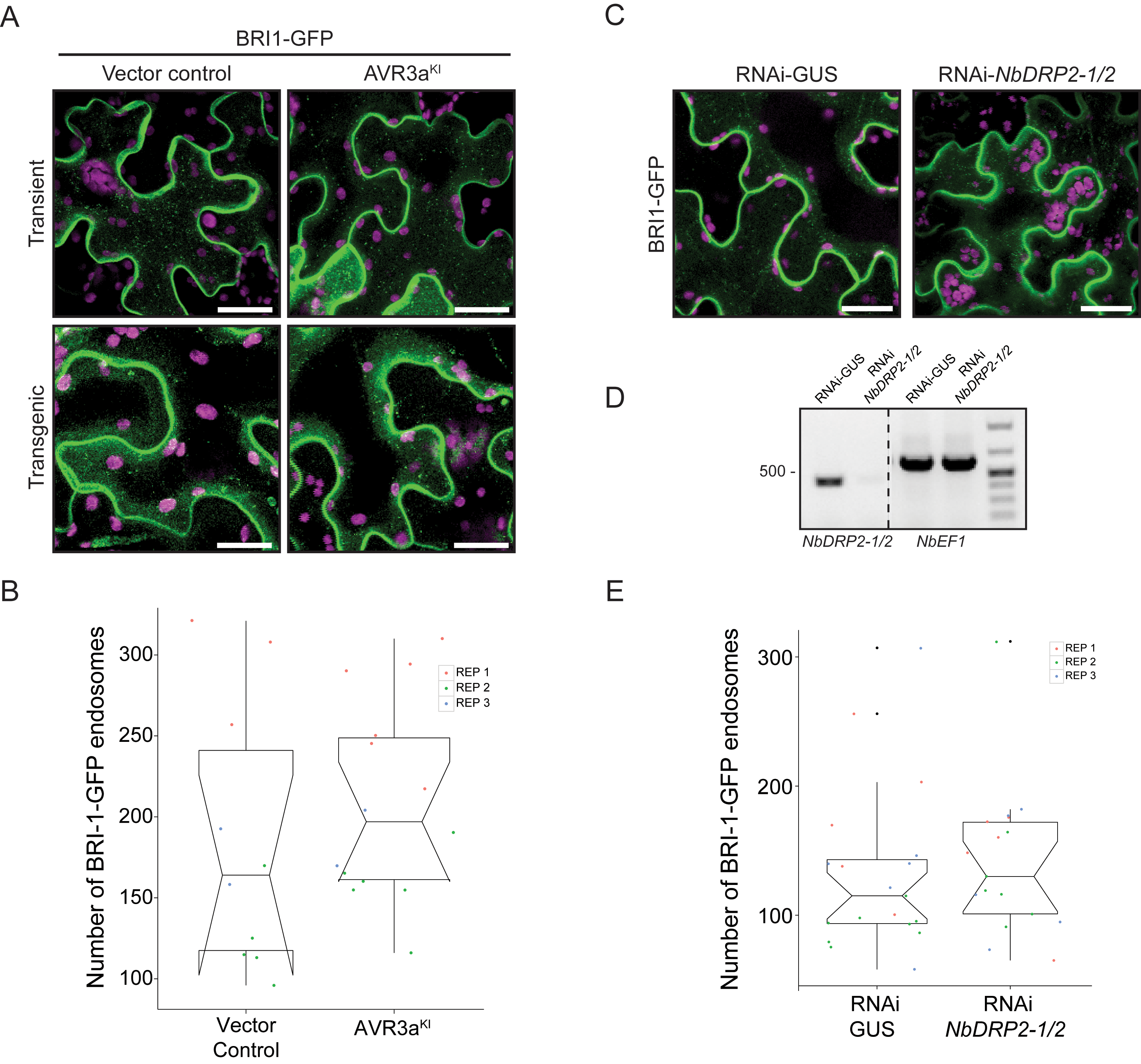

Supplement: S5 Fig — (A) Confocal microscopy at 2.5 days post infiltration (dpi) in N. benthamiana epidermal leaf cells transiently or stably expressing FLAG-AVR3aKI and infiltrated with BRI1-GFP. AVR3a did not alter the plasma membrane subcellular localization of BRI1 or its constitutive endosomal localization (green dots). Bar = 50 μm. Plastids auto-fluorescence (purple) is shown. (B) Quantification of the effect of AVR3a on AtBRI1 endocytosis. Scatter plots show the number of BRI1-GFP endosomes per total image area from 3 independent biological experiments. Vector control (ΔGFP) n = 10, FLAG-AVR3aKI n = 14. No statistical difference was found as assessed by Wilcoxon-Mann-Whitney Test. P = 0.3. (C) BRI1-GFP was co-expressed with a hairpin-silencing construct for NbDRP2–1/2 or the vector RNAi-GUS and confocal imaging was done at 2.5 dpi. Reduced levels of expression of NbDRP2–1/2 did not change BRI1-GFP intracellular vesicle-like (green dots) or plasma membrane localization. Bar = 50 μm. Plastids auto-fluorescence (purple) is shown. All images are a maximum projection of 21 slices taken at 1-μm intervals. Same confocal settings were used to acquire all images. (D) Validation of NbDRP2–1/2 silencing by RT-PCR in leaf-discs collected from the same leaves used for microscopy. (E) Quantification of the effect of RNAi-NbDRP2–1/2 on AtBRI1 endocytosis. Scatter plots show the number of BRI1-GFP endosomes per total image area from 3 independent biological experiments. RNAi-GUS n = 27, RNAi-NbDRP2–1/2 n = 27. No statistical difference was found as assessed by Student’s t test. P = 0.1. (TIF) [file pone.0137071.s005.tif]

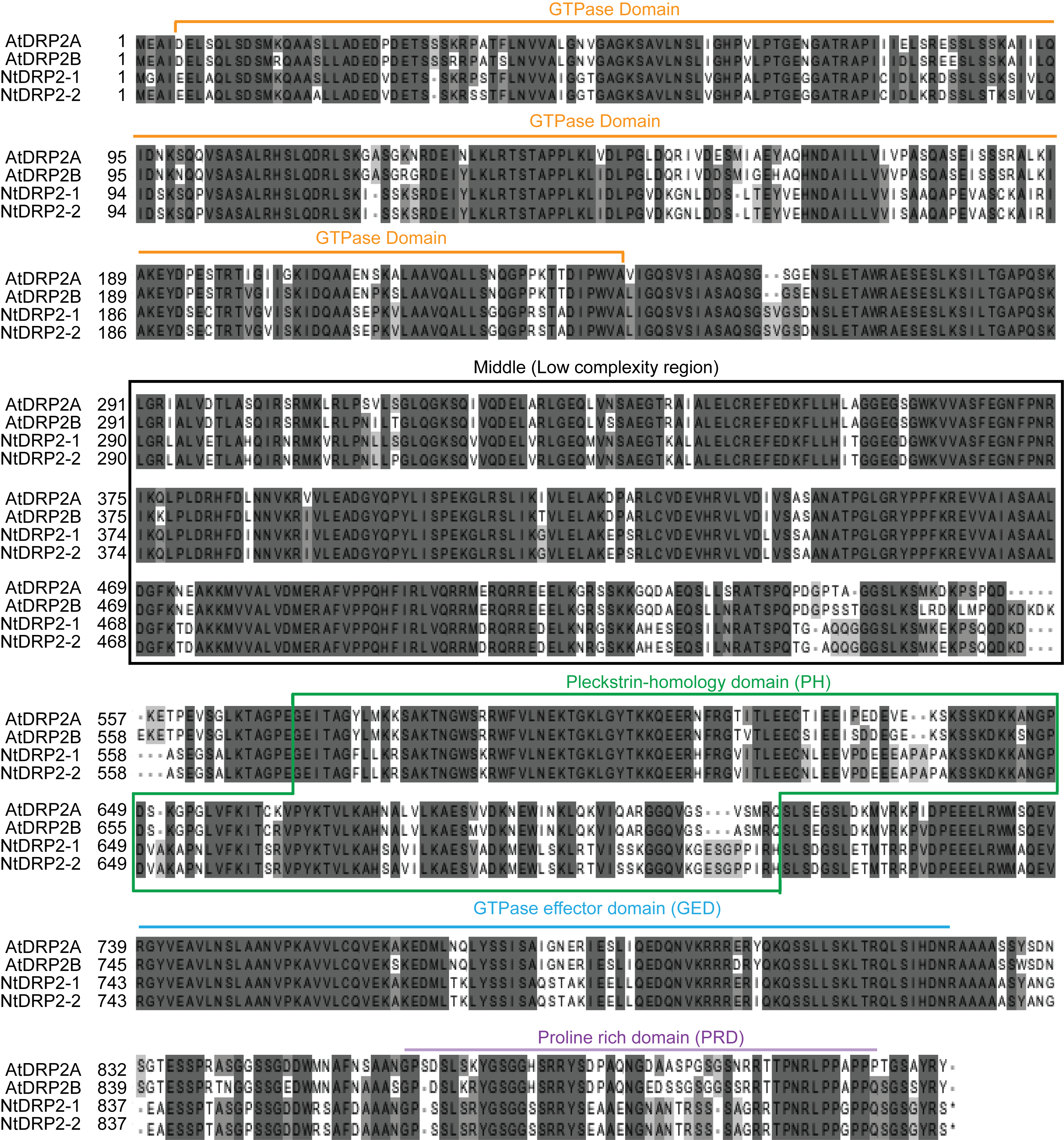

Supplement: S6 Fig — ClustalW alignment shows Arabidopsis DRP2A and DRP2B dynamin GTPase proteins and homologs in N. tabacum. Amino acid residues are shaded dark grey if identical and a lighter shade of grey if similar. Sequences were viewed in Jalview. Full-length sequences were used for the alignment. Canonical domains previously described for large dynamin GTPase are shown. (TIF) [file pone.0137071.s006.tif]

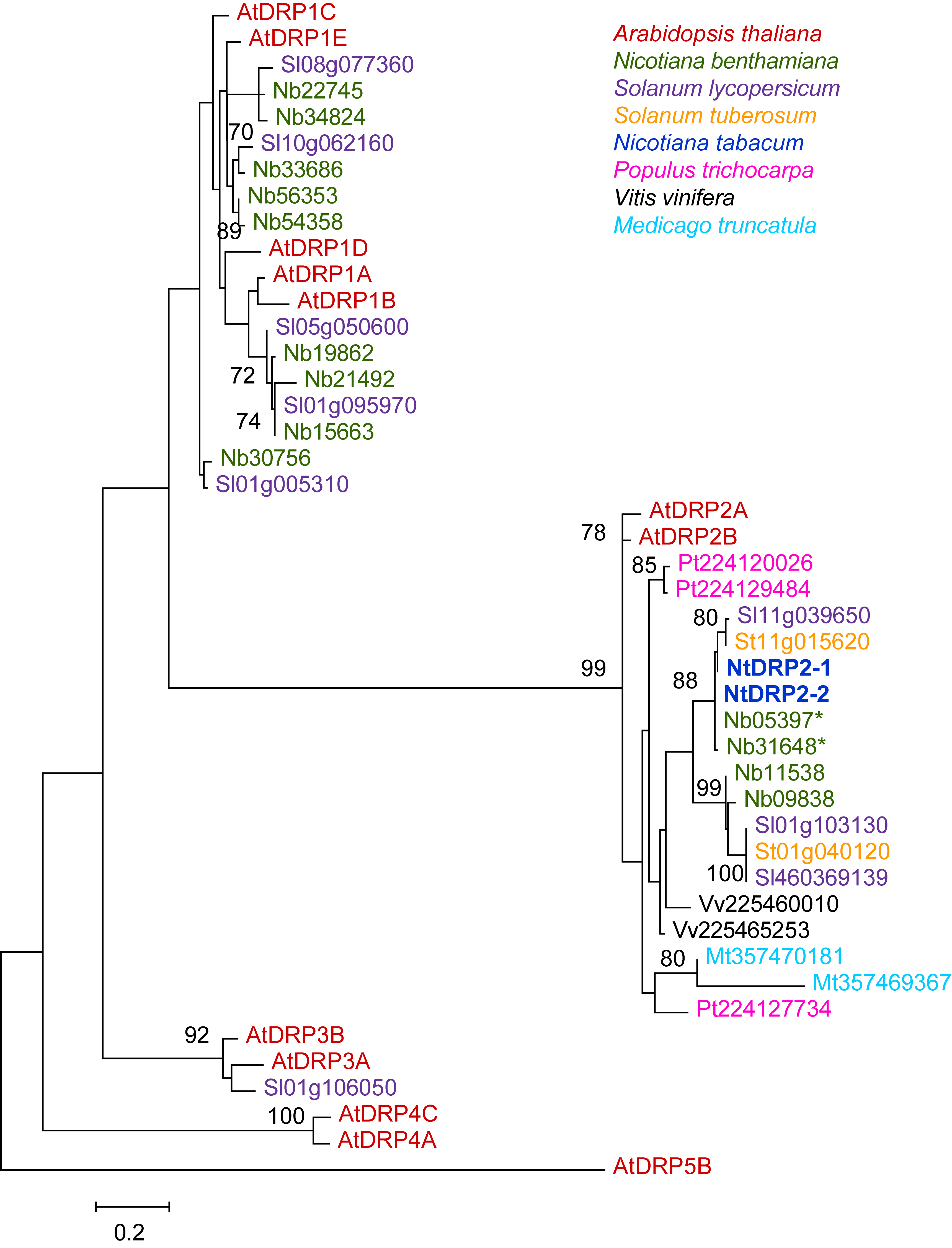

Supplement: S7 Fig — Phylogenetic tree of dynamin-related proteins (DRP) from A. thaliana (red), N. benthamiana (green), tomato (purple), potato (yellow), N. tabacum (dark blue), P. trichocarpa (pink), V. vinifera (black) and M. truncatula (light blue). The conserved GTPase-domain of DRP proteins was aligned by MUSCLE and analyzed with RAxML to construct a phylogenetic tree using the maximum likelihood method. Branch length represents the estimated genetic distance. Bootstrap values for 500 replicates are shown. The sequence identifiers are from the Solgenomics, NCBI and Arabidopsis database. Green asterisks indicate the homologs of Arabidopsis DRP2A and DRP2B in N. benthamiana targeted by silencing in this study. (TIF) [file pone.0137071.s007.tif]

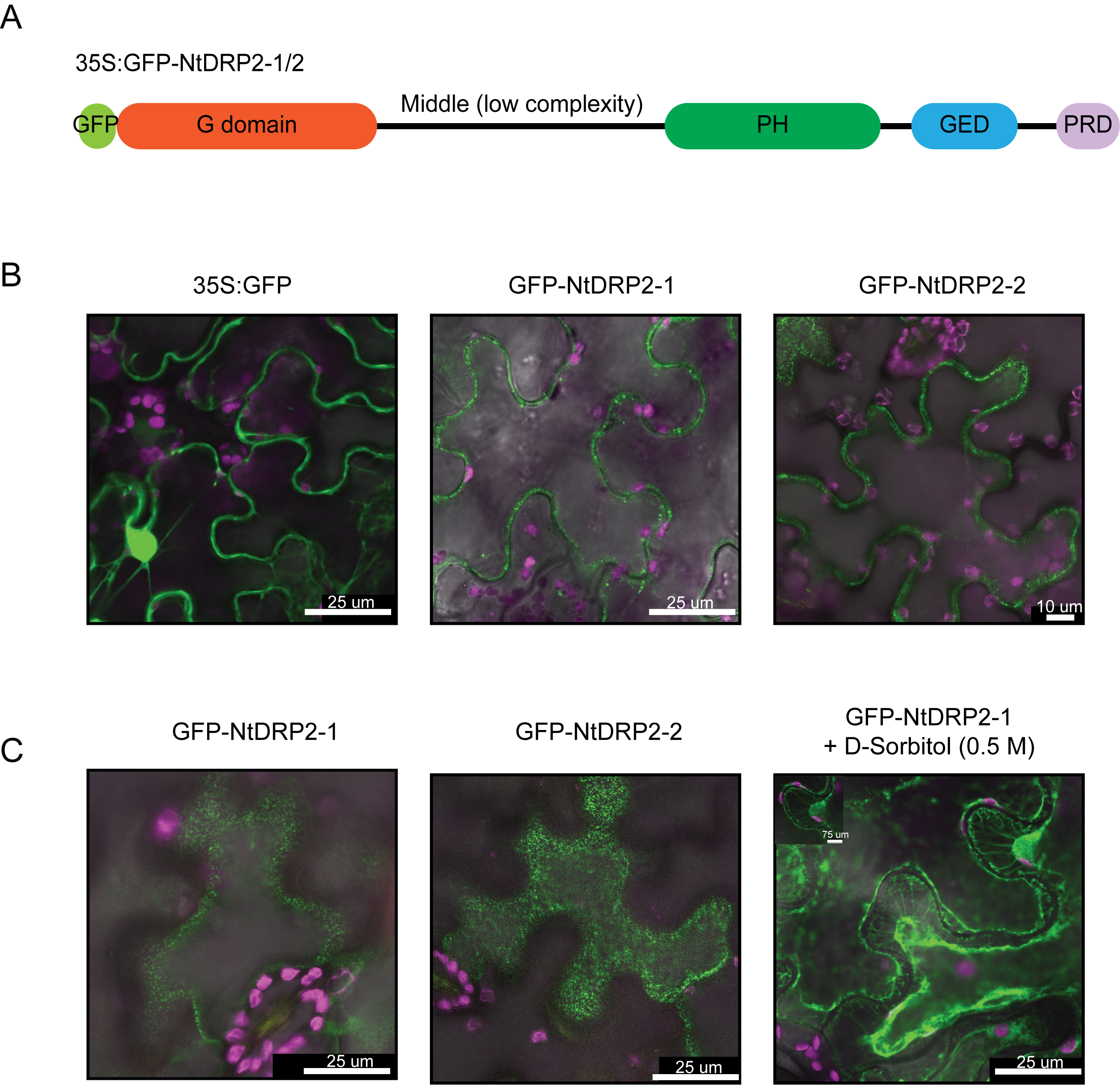

Supplement: S8 Fig — (A) Schematic representation of N. tabacum dynamin proteins (NtDRP2–1 and NtDRP2–2) and their domain organization: GTPase domain (G domain), Pleckstrin homology domain (PH), GTPase effector domain (GED), and a proline-rich domain (PRD). (B, C) Confocal microscopy in N. benthamiana pavement cells of Agrobacterium-mediated expressing NtDRP2–1/2 GFP fusions. GFP-NtDRP2–1 or GFP-NtDRP2–2 primarily localized to the plasma membrane as confirmed by plasmolysis (C), far right end. (C) GFP-NtDRP2–1 and GFP-NtDRP2–2 also localized in punctuate, small vesicle-like structures. Scale bar values are shown in each picture. Representative confocal images were taken at 2.5 days post infiltration. Plastids auto-fluorescence (purple) is shown. (TIF) [file pone.0137071.s008.tif]

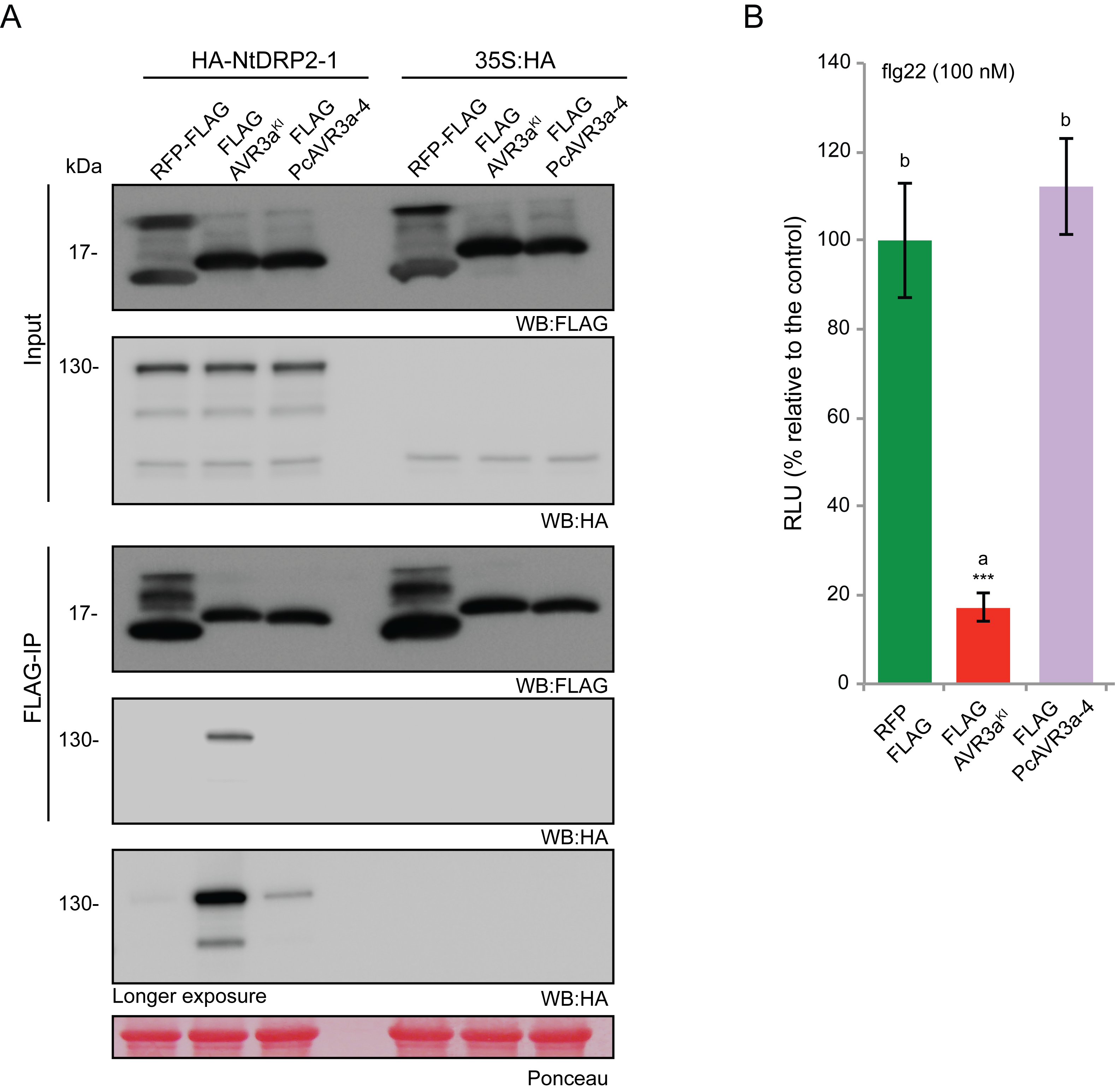

Supplement: S9 Fig — (A) Phytophthora capsici AVR3a-4 effector weakly co-immunoprecipitates with NtDRP2–1 in planta. HA-NtDRP2–1 was transiently co-expressed with FLAG-AVR3aKI, FLAG-PcAVR3a-4 or FLAG-RFP (control) in N. benthamiana and immunoprecipitated with anti-FLAG antiserum (SIGMA). Immunoprecipitates and total protein extracts were immunoblotted with the appropriate antisera. (B) Oxidative burst triggered by 100 nM flg22 in N. benthamiana agroinfiltrated with members of the Avr3a family FLAG-AVR3aKI, FLAG-PcAVR3a-4 or FLAG-RFP (control). ROS production was measured in relative light units (RLU) over time and depicted relative to the total ROS burst of the control. Values are average ± SE (n = 16). Statistical significance was evaluated in comparison to the control by one-way ANOVA followed by TukeyHSD test. *** P < 0.001. Experiment was repeated 3 times with similar results. (TIF) [file pone.0137071.s009.tif]

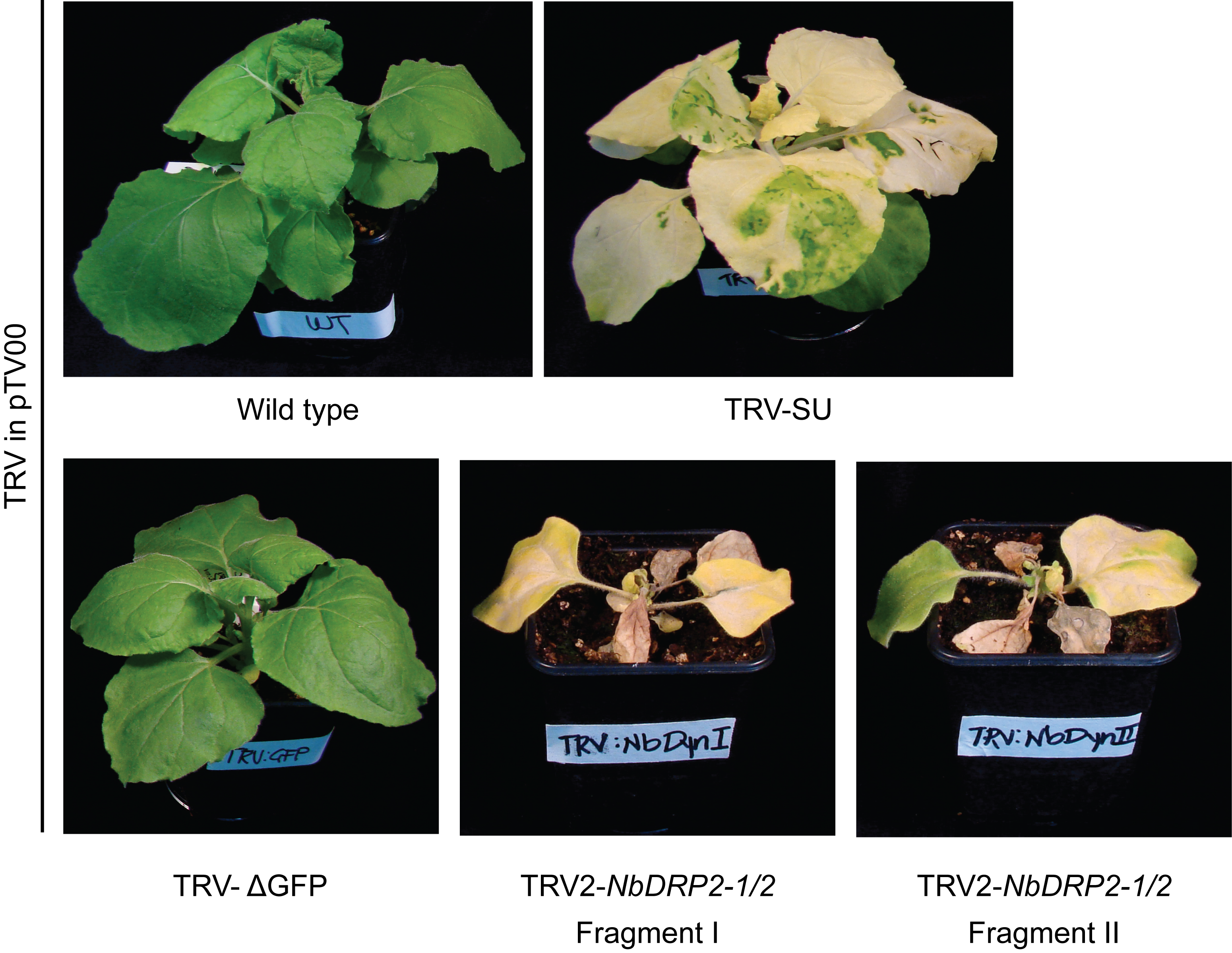

Supplement: S10 Fig — N. benthamiana plants were silenced using tobacco rattle virus vectors harboring a partial sequence of NbDRP2–1 (TRV::NbDRP2–1/2 Fragment I or TRV::NbDRP2–1/2 Fragment II) or an empty cloning site (TRV::GFP). Pictures were taken 2.5 weeks after the initial infiltration with the silencing constructs. (TIF) [file pone.0137071.s010.tif]

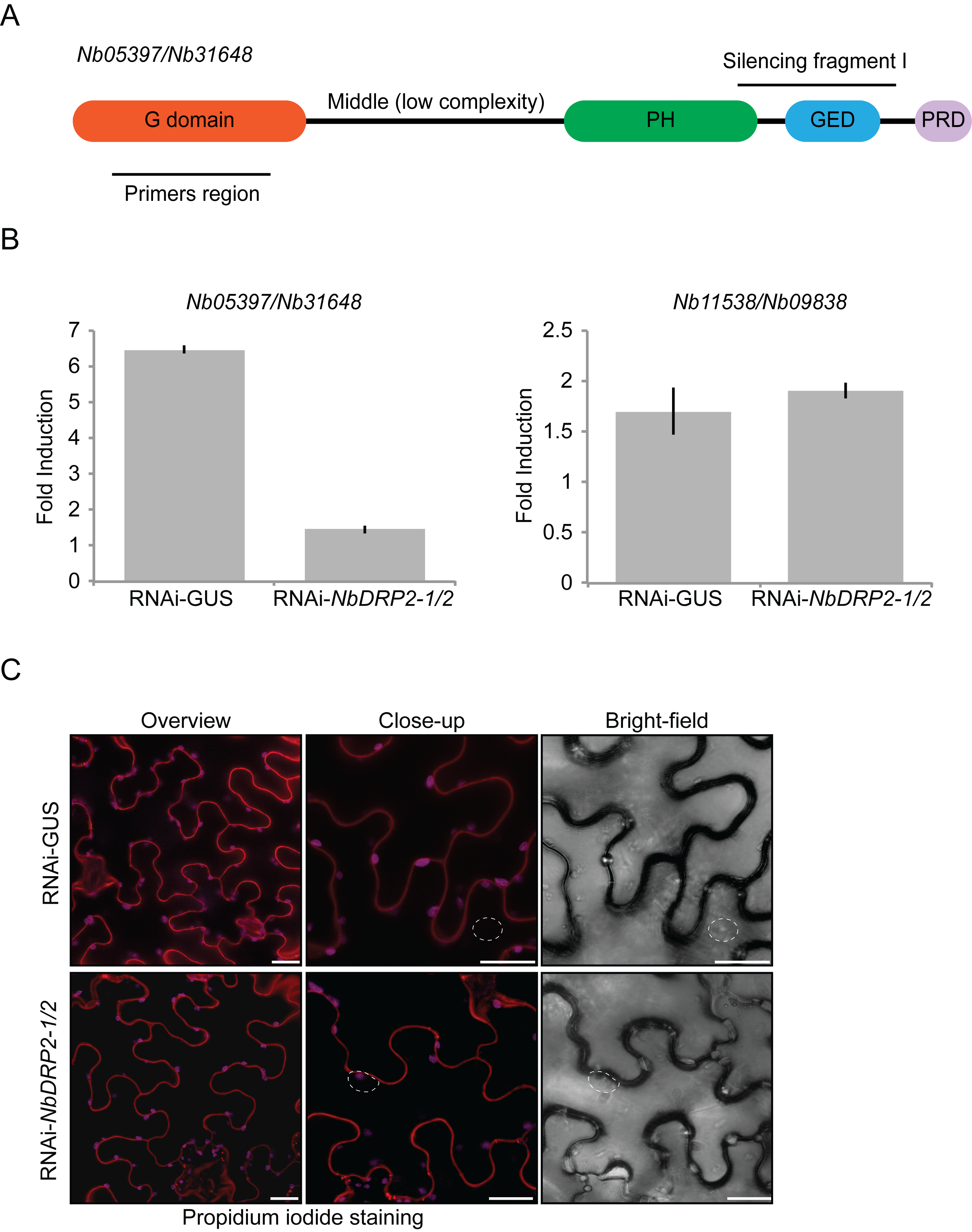

Supplement: S11 Fig — (A) Schematic representation of NbDRP2–1/2 (Nb05397 and Nb31648) showing the canonical dynamin domains and the sequence region targeted by the silencing fragment. (B) Constructs carrying a hairpin plasmid (pHellsgate 8) targeting NbDRP2–1/2 or GUS (RNAi-NbDRP2–1/2 and RNAi-GUS, respectively) were infiltrated in N. benthamiana and the expression of N. benthamiana homologs of AtDRP2A/B was assessed by qRT-PCR at three days post silencing. The silencing fragment targeting part of the pleckstrin homology domain (PH, green) and the GTPase effector domain (blue) specifically knocks down the expression of Nb05397 and Nb31648 (NbDRP2–1 and NbDRP2–2) but not the expression of Nb11538 or Nb09838. Gene expression was normalized to NbEF1α. (C) The NbDRP2–1/2 genes were transiently silenced in N. benthamiana and at three days post silencing, the epidermal cells were stained for 5 minutes with a solution of propidium iodide (PI). Auto-fluorescence of chloroplasts (purple) is shown. The absence of PI fluorescence in the cell nucleus (dashed white lines) indicates that the cells are still viable. Bar = 25 μm. (TIF) [file pone.0137071.s011.tif]

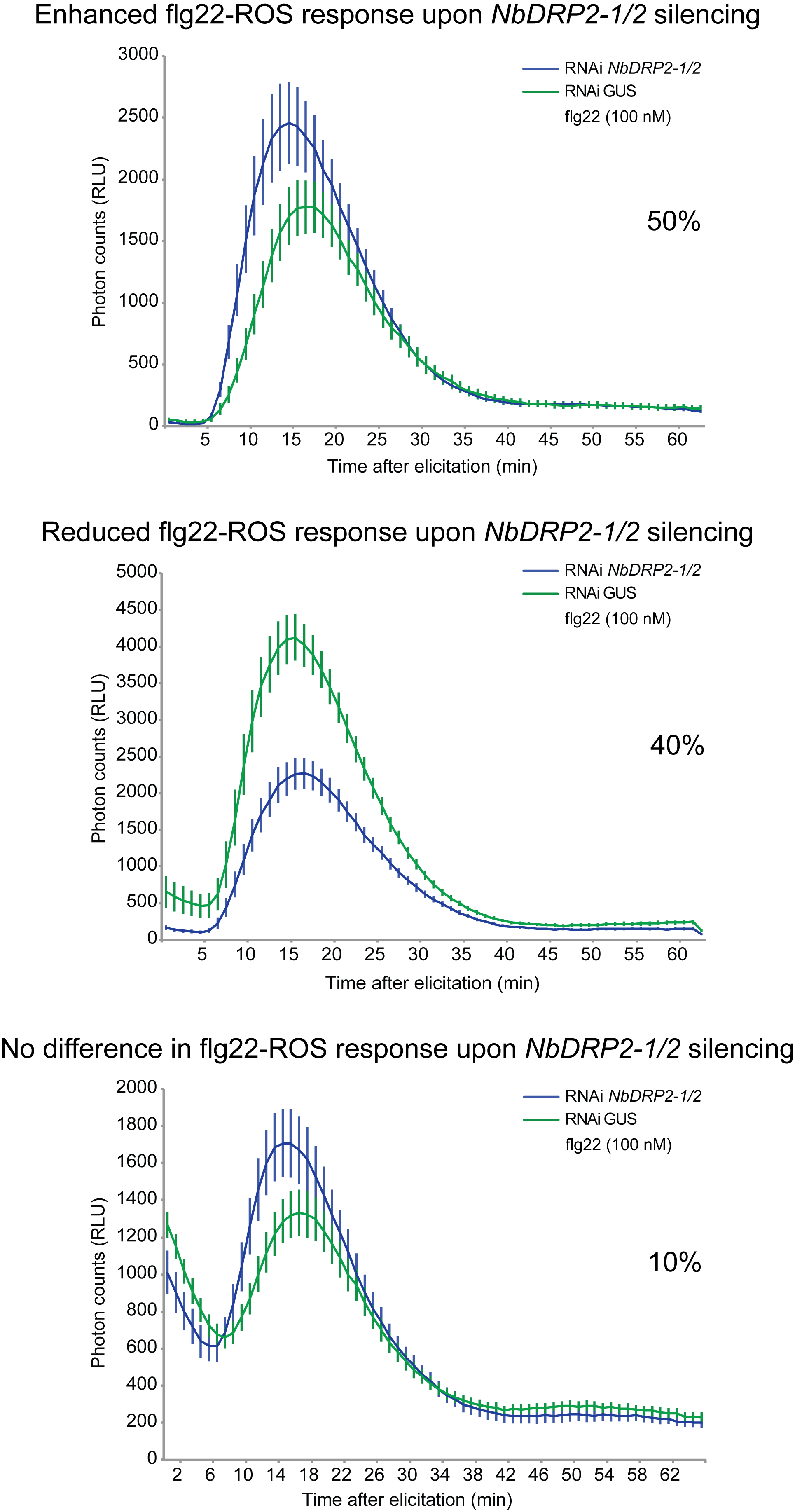

Supplement: S12 Fig — (TIF) [file pone.0137071.s012.tif]
